# Eradication of Metastatic Renal Cell Carcinoma after Adenovirus-Encoded TNF-Related Apoptosis-Inducing Ligand (TRAIL)/CpG Immunotherapy

**DOI:** 10.1371/journal.pone.0031085

**Published:** 2012-02-01

**Authors:** Lyse A. Norian, Timothy P. Kresowik, Henry M. Rosevear, Britnie R. James, Timothy R. Rosean, Andrew J. Lightfoot, Tamara A. Kucaba, Christopher Schwarz, Christine J. Weydert, Michael D. Henry, Thomas S. Griffith

**Affiliations:** 1 Department of Urology, The University of Iowa Carver College of Medicine, Iowa City, Iowa, United States of America; 2 Microbiology, Immunology, and Cancer Biology Program, University of Minnesota, Minneapolis, Minnesota, United States of America; 3 Interdisciplinary Graduate Program in Immunology, The University of Iowa Carver College of Medicine, Iowa City, Iowa, United States of America; 4 Department of Urology, University of Minnesota, Minneapolis, Minnesota, United States of America; 5 Department of Physiology, The University of Iowa Carver College of Medicine, Iowa City, Iowa, United States of America,; French National Centre for Scientific Research, France

## Abstract

Despite evidence that antitumor immunity can be protective against renal cell carcinoma (RCC), few patients respond objectively to immunotherapy and the disease is fatal once metastases develop. We asked to what extent combinatorial immunotherapy with Adenovirus-encoded murine TNF-related apoptosis-inducing ligand (Ad5mTRAIL) plus CpG oligonucleotide, given at the primary tumor site, would prove efficacious against metastatic murine RCC. To quantitate primary renal and metastatic tumor growth in mice, we developed a luciferase-expressing Renca cell line, and monitored tumor burdens via bioluminescent imaging. Orthotopic tumor challenge gave rise to aggressive primary tumors and lung metastases that were detectable by day 7. Intra-renal administration of Ad5mTRAIL+CpG on day 7 led to an influx of effector phenotype CD4 and CD8 T cells into the kidney by day 12 and regression of established primary renal tumors. Intra-renal immunotherapy also led to systemic immune responses characterized by splenomegaly, elevated serum IgG levels, increased CD4 and CD8 T cell infiltration into the lungs, and elimination of metastatic lung tumors. Tumor regression was primarily dependent upon CD8 T cells and resulted in prolonged survival of treated mice. Thus, local administration of Ad5mTRAIL+CpG at the primary tumor site can initiate CD8-dependent systemic immunity that is sufficient to cause regression of metastatic lung tumors. A similar approach may prove beneficial for patients with metastatic RCC.

## Introduction

Patients with metastatic renal cell carcinoma (RCC) face a dismal prognosis and have limited therapeutic options. Median survival in a recent cohort was only 1.5 years with fewer than 10% of patients surviving to five years [1]. Immunotherapy with high dose IL-2 has a 20% response rate, including a 5–10% complete response rate, but is poorly tolerated and has significant side effects that limit its use to specialized centers with highly selected patients [2], [3], [4], [5]. While newer treatments, such as the multikinase inhibitors sunitinib and sorafenib, show small improvements in survival, complete responses are rare with these agents [6]. A treatment that could deliver durable, complete responses without the rigors of high dose IL-2 would be a major advance for patients with this deadly disease.

TNF-related apoptosis-inducing ligand (TRAIL) is a member of the Tumor Necrosis Factor family that has the ability to induce apoptosis in malignant cells, while largely sparing untransformed, normal tissues [7], [8], [9], [10]. TRAIL ligation of cognate death-inducing receptors (TRAIL-R1 and TRAIL-R2 in humans, DR5 in mice) [11] triggers apoptotic death in tumor cells, thereby increasing the amount of tumor cell antigen potentially available for uptake and processing by local antigen-presenting cells (APCs) [10]. Typically, phagocytosis of apoptotic bodies by APCs results in immune tolerance rather than protective immunity [12]. Therefore, to initiate protective anti-tumor immunity, APCs processing TRAIL-generated apoptotic tumor cells need to receive a separate stimulatory signal. CpG oligodeoxynucleotides contain unmethylated CG motifs that bind to toll-like receptor 9 (TLR9), activating APCs and increasing their MHC and co-stimulatory molecule expression, and cytokine production [13], [14]. As a result, co-administration of CpG with TRAIL provides the stimulatory signal APCs need to initiate protective immunity to tumor-derived antigens. Both TRAIL and CpG have shown minimal toxicity in Phase I clinical trials, making them excellent candidates for antitumor immunotherapies [9], [15].

We showed previously that intratumoral co-administration of a replication-deficient adenovirus encoding murine TRAIL (Ad5mTRAIL) plus CpG1826 resulted in enhanced CD8 T cell-mediated antitumor immunity and clearance of localized subcutaneous (s.c.) Renca tumors in mice [16]. In that model, therapeutic administration of Ad5mTRAIL+CpG led to a systemic memory CD8 T cell response that protected mice from subsequent Renca re-challenge. In contrast, CD4 T cells were largely suppressive, and impaired both Ad5mTRAIL+CpG efficacy and CD8 T cell proliferation. The induction of a humoral immune response to intratumoral Ad5mTRAIL+CpG therapy was not investigated. Therefore, despite its encouraging results, our prior study had several important limitations. First, as the numbers and types of APCs differ substantially from one anatomic location to another, demonstrated Ad5mTRAIL+CpG efficacy in a localized s.c. model of RCC provided little evidence that the therapy would be effective in a more physiologically relevant orthotopic RCC model, where CpG would have to activate limited numbers of renal APCs. Furthermore, as the primary clinical application of immunotherapy is in the treatment of advanced cancers that have already disseminated, it was important for us to evaluate the efficacy of Ad5mTRAIL+CpG in a pre-clinical model of metastatic RCC.

Recent evidence in a small number of clinical cases suggests that T cell-mediated eradication of metastatic RCC in patients may be feasible [17], [18], [19]. To determine the extent to which local administration of Ad5mTRAIL+CpG at the primary tumor site would stimulate T cell-mediated eradication of metastatic RCC, we developed a murine model based on intrarenal (IR) injections of Renca tumor cells engineered to express Luciferase. Renca cells injected IR develop into primary renal tumors that spontaneously metastasize to the lungs [20], [21], [22]. Luciferase expression in tumor cells allows for the quantitative analysis of primary and metastatic tumor growth in live animals over time. As the lungs are one of the primary metastatic sites in patients with advanced RCC [23], this model provides a clinically relevant means of assessing the immunotherapeutic efficacy of Ad5mTRAIL+CpG against metastatic RCC. We demonstrate here that Ad5mTRAIL+CpG, given at the primary renal tumor site, leads to infiltration of tumor-bearing kidneys by effector CD4 and CD8 T cells. It also leads to increased infiltration of CD4 T cells and CD8 T cells into the lungs. Elimination of both primary and metastatic tumors ensues and is mediated primarily by CD8 T cells. In addition, Ad5mTRAIL+CpG induces splenomegaly and a humoral response characterized by increased serum levels of total IgG, anti-adenoviral IgG, and anti-dsDNA without progression to autoimmunity. These results demonstrate the feasibility of using a T cell-stimulatory immunotherapy for the treatment of metastatic RCC, and suggest that Ad5TRAIL+CpG as a therapeutic approach may be an efficacious, yet well-tolerated option for patients with advanced disease.

## Methods

### Mice/Ethics Statement

Female BALB/c mice (7–8 wk old) were purchased from the National Cancer Institute or Harlan Laboratories. All animal procedures were approved by the Institutional Animal Care and Use Committee at The University of Iowa (ACURF #0912282).

### Cell lines

The murine renal adenocarcinoma cell line, Renca, was obtained from Dr. Robert Wiltrout (National Cancer Institute, Frederick, MD), and was authenticated in 2010 by microsatellite marker analysis (Research Animal Diagnostic Laboratory, Columbia, MO). Renca cells were maintained in Complete RPMI as previously described [16]. Renca-Luc is a variant that stably expresses firefly Luciferase; it was generated via retroviral transduction as described [24], [25]. Renca-Luc cells were maintained in Complete RPMI supplemented with 0.05 µg/ml puromycin.

### Tumor challenge

For IR tumor challenge, a skin incision was made on the left flank, and 2×10^5^ Renca or Renca-Luc cells were injected through the intact peritoneum into the left kidney. On d 7 following tumor challenge, mice were re-injected in the same kidney with either sterile PBS, or 10^9^ pfu of replication-deficient Ad5mTRAIL that encodes a membrane-bound version of full-length murine TRAIL protein with or without 100 µg CpG1826 (Coley Pharmaceuticals, Wellesley, MA), in a 100 µl volume. Mice were sacrificed between d 21–25, when untreated renal tumors were palpable. In some experiments, CD4^+^ or CD8^+^ cells were depleted *in vivo* via i.p. injection of either 100 µg/mouse GK1.5 (CD4) or 53.6.72 (CD8) antibodies given on d 4, 6, 7, 14, and 21. Depletion efficacy was monitored by flow cytometric analysis of CD4 and CD8 staining of spleen samples in test mice; depletion of both T cell populations was found to be greater than 90% (data not shown). In some mice, s.c. tumor challenges were performed by injecting 2×10^5^ Renca-Luc cells into the right hind flank; s.c. tumors were allowed to grow for 37 d, at which time bioluminescent imaging (BLI) was performed on live mice and excised lungs from euthanized animals.

### Bioluminescent imaging (BLI)

BLI was done using an IVIS 200 (Caliper Life Sciences, Hopkinton, MA) as described [24], [25]. Briefly, 10 min prior to imaging, mice were injected i.p. with 100 µl of a 15 µg/ml solution of D-Luciferin (GoldBio.com, St. Louis, MO), then anesthetized via inhalation of oxygenated isoflurane. Live mice were imaged for 1 min. Excised organs were imaged separately with a 5 min exposure. Photon flux (photons/second) was calculated within a defined region of interest using Living Image software (Version 2.5).

### Flow cytometry

Tumors, lungs, and spleens were harvested, manually disrupted, then digested for 15–30 min in HBSS containing 0.56 Wuensch units/ml of Liberase Blendzyme 3 (Roche, Branford, CT) and 0.15 µg/ml DNAse I (Sigma, St. Louis, MO), and prepared as described to generate single cell suspensions [26]. Cells were stained with combinations of the following antibodies, and results acquired using multi-parameter flow cytometry on a BD LSR II (BD Biosciences, San Diego, CA) then analyzed with FlowJo software. An expanded FS/SSC gate was used to encompass not only resting and blasted lymphocytes, but larger DC and more granular macrophages, as appropriate. Doublets were excluded by standard FSC-A/FSC-W gating. Dead cells were excluded via Hoechst positivity. For DC: CD11c-biotin, streptavidin-APC/Cy7, CD11b-PE/Cy7, Gr1-APC, I-A^d^-PE, CD4-FITC; for T cells: CD3-PE, CD8-APC, CD4-PE/Cy7, CD44-biotin, streptavidin APC/Cy7; for macrophages: CD11b-PE/Cy7, for B cells: B220- A488; for NK cells: DX5-FITC. Antibodies were from eBioscience (San Diego, CA) or BioLegend (San Diego, CA).

### Histology and microscopy

Excised kidneys were fixed in 10% Neutral Buffered Formalin, embedded in paraffin, sectioned at a thickness of 8 microns, then stained with Hematoxylin and Eosin. Sample processing and staining was performed by the University of Iowa Department of Pathology Histology Core facility. Photomicrographs were taken on an Olympus BX61 light microscope using the 4× or 10× objective and CellSens Dimension software (Olympus Corp, Cedar Valley, PA). Images were processed using Preview v4.2 software (Apple Inc., Cupertino, CA).

### ELISA analysis of total IgG, anti-adenoviral IgG, and anti-dsDNA Ab

Mice (3–10 per indicated treatment group) were bled on d 12, 20, and 48 after IR Renca tumor challenge. Serum was obtained by centrifugation and frozen until use. Total IgG was measured using a Mouse IgG ELISA kit (Immunology Consultants Laboratory, Newberg, OR) according to the manufacturer's specifications. Measurement of anti-adenoviral IgG [27] was performed by coating a 96-well microtiter plate with 10^9^ Ad5mTRAIL particles/well in 100 µl sodium bicarbonate overnight at 4°C. Wells were then washed and blocked in 100 µl PBS containing 3% BSA and 0.01% Tween-20. Afterwards, the plate was incubated 2 h at RT with 100 µl diluted plasma or mouse adenovirus IgG1. Wells were washed, 100 µl HRP-conjugated goat anti-mouse IgG Fc was added and incubated 2 h at RT. Wells were washed and 100 µl TMB peroxidase substrate was added. The reaction was stopped by adding 100 µl 1 M H_2_SO_4_, and absorbance was measured at 450 nm. Anti-dsDNA IgG was measured using a mouse anti-dsDNA ELISA Kit (Shibayagi Co., Japan) according to the manufacturer's specifications.

### Statistics

Statistical significance was determined as *p*<0.05 for specified sets of data via unpaired student t-test with Welch's correction for unequal variances. Data were analyzed using Prism software (GraphPad Software Inc., La Jolla, CA).

## Results

### Aggressive primary tumors and spontaneous lung metastases form following IR injections of Renca tumor cells

The Renca cell line is derived from a spontaneously arising renal adenocarcinoma of BALB/c mice [28], and is widely used to model RCC. In many studies, Renca cells are injected s.c. to produce localized tumors or intravenously (i.v.) to produce experimental lung metastases [29], [30], [31], [32], [33]. We had previously shown that intratumoral administration of Ad5mTRAIL plus CpG1826 could lead to the eradication of localized s.c. Renca tumors in mice [16]. In the current study, we evaluated the efficacy of an Ad5mTRAIL+CpG combinatorial immunotherapy in a more physiologically relevant orthotopic Renca model, where IR tumor challenge gives rise to spontaneous lung metastases [20], [22], [34].

**Figure 1:**
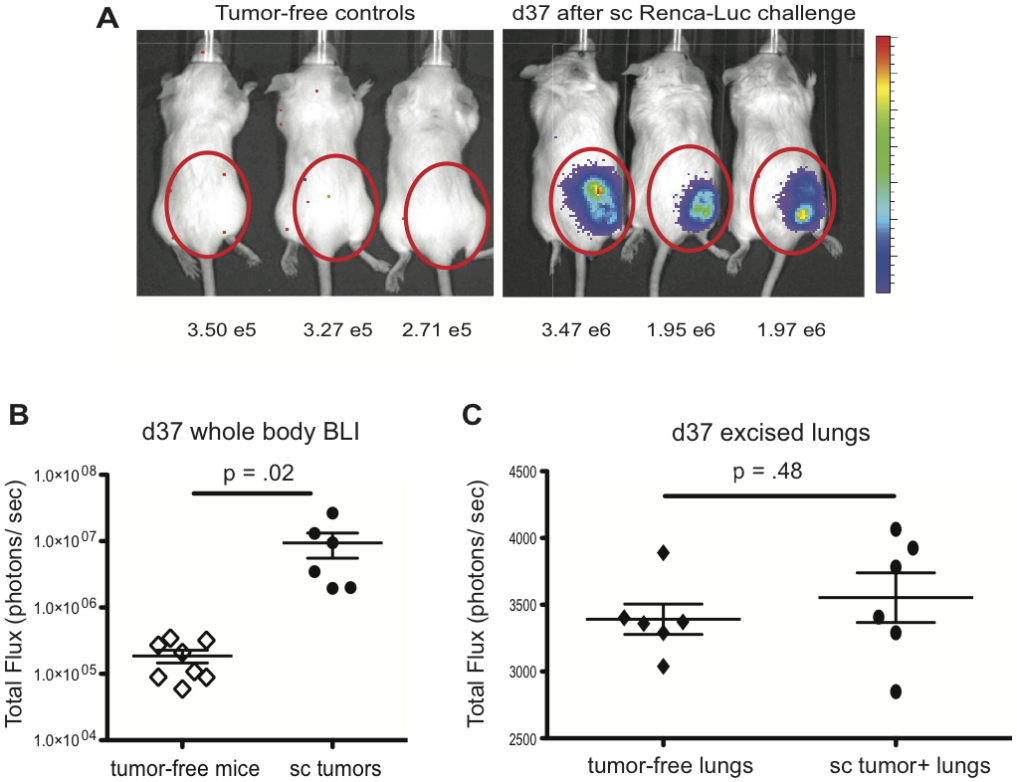
Renca s.c. tumor challenge produces only local tumor growth. (A) Renca-Luc cells were injected s.c. and whole-body bioluminescent images were taken on d 37. Shown are 3 tumor-free controls and 3 tumor-bearing mice. (B) Total light flux values acquired via BLI on d 37 for individual tumor-bearing mice (n = 6). The mean background light flux for 8 tumor-free mice is also shown. Data are cumulative from 2 independent experiments. (C) Total light flux values for individual excised lungs taken from tumor-free or tumor-bearing mice, as indicated.

To verify that our previous s.c. tumor challenge route did, in fact, produce only localized tumors without metastases, we injected a firefly Luciferase-expressing Renca variant (Renca-Luc) s.c. into BALB/c mice and allowed it to grow unchecked through d 37. At this time, whole-body BLI was performed on live tumor-challenged and tumor-free control mice (Fig. 1A and B). Light flux values in the flank were significantly greater than the background flux emitted by tumor-free control mice, indicating positive tumor growth. However, no disseminated tumor growth was evident. We then euthanized mice and excised the lungs to permit a more sensitive evaluation via BLI. The lungs of s.c. tumor-challenged mice had similar light emission to excised lungs from tumor-free control mice, indicating an absence of tumor growth in this site (Fig. 1C). Therefore, Renca cells injected s.c. into the flank do not metastasize from the injection site, a finding that is in agreement with a recent study by Westwood et al. [20].

**Figure 2:**
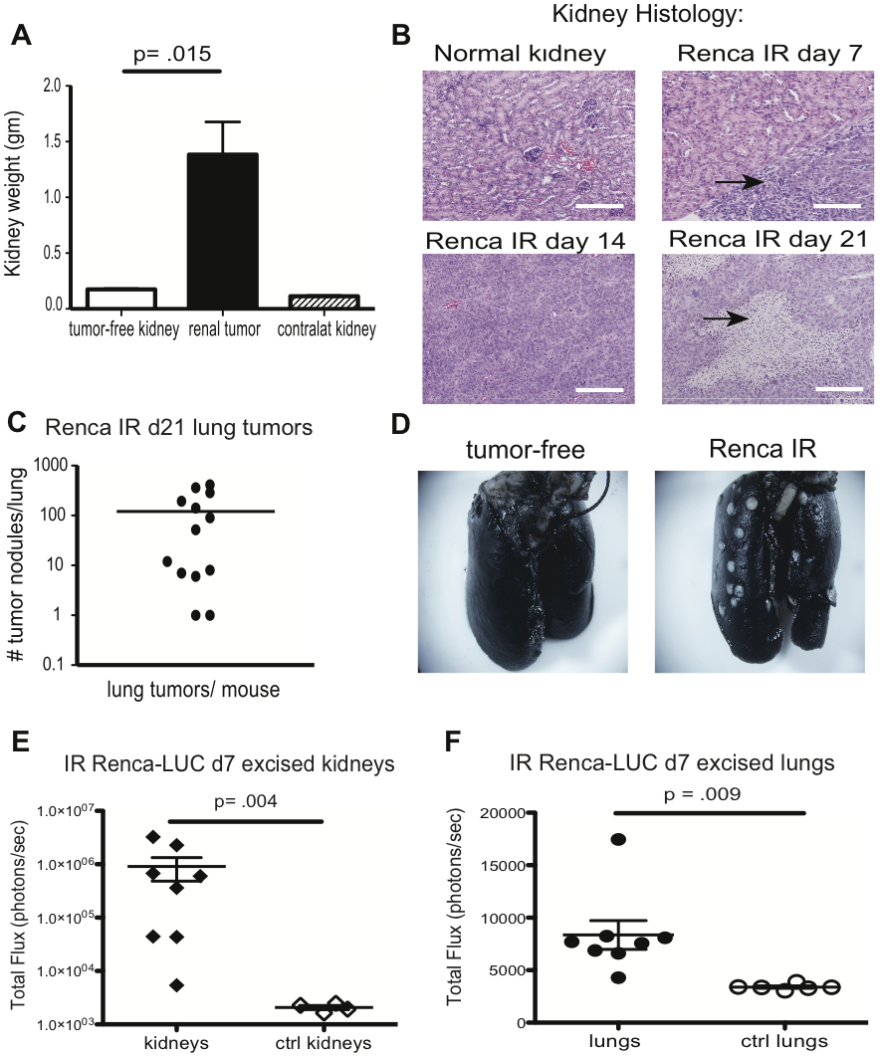
Orthotopic injection of Renca tumor cells leads to aggressive primary tumors and spontaneous lung metastases. (A) Parental Renca cells were injected IR, and weights of excised kidneys in grams (gm) were recorded on d 23. Representative data from >5 independent experiments are shown (n = 5 mice per group). (B) H&E-stained sections from one normal tumor-free kidney, or tumor-bearing kidneys harvested at the times indicated. Areas of dense purple staining indicate renal tumors. Scale bar = 200 µm. (C, D) Quantitation of visible lung tumors from 13 individual mice. Lungs were inflated with India Ink *en bloc* to allow visualization of white lung tumor nodules. (E, F) Total light flux values for individual excised kidneys (E) and lungs (F) taken from mice on d 7 after IR Renca-Luc challenge or tumor-free (ctrl) mice.

We then proceeded to evaluate the ability of Ad5mTRAIL+CpG to eliminate metastatic lung tumor growth when administered intra-renally (IR) at the site of established primary kidney tumors. To characterize orthotopic renal tumor formation in the absence of immunotherapy, parental Renca cells were injected directly into the left kidney of each mouse, and tumor growth was assessed via measurement of excised kidney weights on d 23. Large renal masses were evident in challenged kidneys, while contralateral kidneys remained grossly unaltered (Fig. 2A). Primary tumor growth was rapid, and resulted in histologically detectable tumor formation as early as d 7 post-challenge; by d 14 normal architecture was lost in nearly the entire kidney, and necrotic areas within renal tumors were observed by d 21 (arrows, Fig. 2B).

In humans, advanced renal tumors metastasize to the lungs [23]. To determine if lung metastases had developed in mice challenged IR with Renca cells, we performed tracheal insufflations of lungs *en bloc* with India Ink. In all challenged mice, tumor nodules were visible on the lung surface by d 21 that could be easily counted (Figs. 2C and 2D), illustrating tumor cell dissemination from the primary IR challenge site and metastatic colonization of lungs. Manual enumeration of surface lung tumors is a frequently performed technique, but this method has several limitations. For example, it may underestimate the total tumor burden per lung, as it cannot identify tumors that form deep within the lung tissue. To complement our use of the parental Renca line, we again used the Renca-Luc variant in the IR tumor challenge of BALB/c mice. Examination of excised kidneys by BLI confirmed the presence of tumor growth in 100% of injected kidneys by d 7 post-challenge – a time when growing tumors were not macroscopically visible (Fig. 2E). The BLI detection of IR Renca-Luc tumor growth at d 7 also corresponded precisely with histologic evaluations performed following parental Renca challenge (Fig. 2B). Of note, we were also able to detect Luciferase-positive tumor cells in the excised lungs of mice at d 7 post-challenge (Fig. 2F). Collectively, these data show that orthotopic tumor challenge of BALB/c mice with Renca or Renca-Luc cells leads to aggressive primary renal tumor outgrowth and the formation of spontaneous lung metastases. As both primary renal tumors and lung metastases are present by d 7 post-IR tumor challenge with Renca cells, this system represents a clinically relevant murine model for evaluating the efficacy of experimental immunotherapies against primary renal and metastatic RCC. BLI of luciferase-expressing tumor cells is beneficial as it permits longitudinal analysis of total, body-wide tumor burdens in live mice. However, since Luciferase may stimulate a certain degree of immune recognition as a foreign protein, we continued to use both Renca-Luc and parental Renca in the following experiments, as each cell type presents unique advantages.

### Local administration of Ad5mTRAIL+CpG immunotherapy leads to regression of primary renal tumors

**Figure 3:**
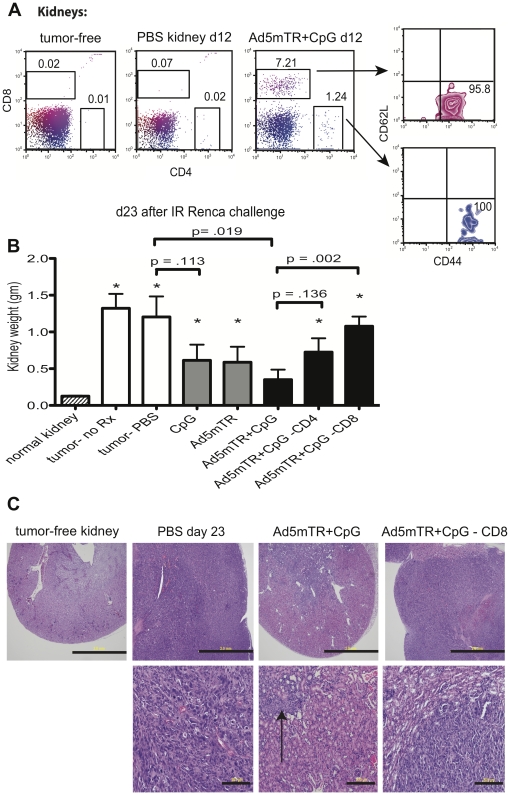
Local combinatorial Ad5mTRAIL+CpG therapy leads to regression of primary renal tumors. (A) Parental Renca cells were injected IR, followed by PBS or Ad5mTRAIL (Ad5mTR) +CpG on d 7. Kidneys were excised on d 12 and analyzed by flow cytometry to assess T cell infiltration. Dot plots show the percentages of live CD8^+^ and CD4^+^ T cells per kidney, as well as expression of CD44 and CD62L on the gated CD8^+^ and CD4^+^ T cells. (B, C) Mice were challenged as in (A) and PBS, Ad5mTR and/or CpG was given on d 7. Tumor-challenged kidneys were excised and weighed on d 23. CD4 and CD8 depletions were performed as described in Methods. Mean values from 4–10 mice per group, combined from 3 individual experiments, are shown. Asterisks indicate *p*<0.05 for that treatment group vs normal, tumor-free kidney weights. Statistical *p* values are shown for PBS vs CpG, PBS vs Ad5mTR+CpG, Ad5mTR+CpG vs Ad5mTR+CpG with CD4 depletion, and Ad5mTR+CpG vs Ad5mTR+CpG with CD8 depletion. For PBS vs CpG alone or Ad5mTR alone *p* = 0.11, for Ad5mTRAIL+CpG –CD8 vs Ad5mTRAIL+CpG –CD4 *p* = .14. (C) In addition, the excised kidneys were processed for H&E staining. Areas of dense purple staining indicate renal tumors. Scale bars in upper panels = 2.0 mm.

One limitation of using s.c. tumor challenges to evaluate the efficacy of experimental immunotherapies is that the frequency and type of APCs differ widely throughout the body. For example, s.c. injection of tumor cells and immunotherapuetics can recruit and activate Langerhans cells [35], [36], but this DC population is not present in the kidney, and therapies that successfully stimulate Langerhans cells may not equivalently stimulate kidney-resident DC populations. To evaluate the efficacy of our combinatorial Ad5mTRAIL+CpG immunotherapy in the IR model of metastatic RCC, mice were challenged with parental Renca cells and treated IR on d 7 with PBS or Ad5mTRAIL+CpG. Kidneys were harvested at d 12 for staining and flow cytometric analysis to determine the extent to which the therapy led to increased T cell infiltration into tumor-bearing kidneys. Administration of Ad5mTRAIL+CpG led to striking increases in the percentage of T cells in tumor-bearing kidneys (Fig. 3A). As shown, approximately 96% of gated CD8 T cells and 100% of gated CD4 T cells were CD44^high^/CD62L^low^, reflecting differentiation to an effector phenotype.

We next asked if the influx of effector T cells into the kidney resulted in renal tumor regression. Mice were challenged IR with parental Renca, then on d 7 were given an IR dose of PBS, CpG alone, Ad5mTRAIL alone, or Ad5mTRAIL+CpG; or were left untreated. On d 23, tumor-challenged kidneys were excised and weighed to evaluate primary tumor growth. Three experimental groups (PBS, CpG, Ad5mTRAIL) showed significantly increased kidney weights as compared to tumor-free control kidneys (Fig. 3B; * *p*<0.05 for a given experimental group vs tumor-free kidneys). In contrast, kidneys from mice that received Ad5mTRAIL+CpG on d 7 remained statistically unchanged in weight through d 23 (Ad5mTRAIL+CpG vs tumor-free, *p* = 0.14). These data indicate that tumors did not grow progressively in mice that received Ad5mTRAIL+CpG on d 7, as they did in mice that received PBS, CpG, or Ad5mTRAIL alone. We also found that IR administration of PBS on d 7 did not alter primary renal tumor outgrowth as compared to mice that received no IR injections on d 7 (PBS vs no Rx, *p* = 0.734), suggesting that physical trauma associated with a second IR injection did not alter the course of tumor outgrowth. Histological evaluations of tumor-challenged kidneys revealed that increased kidney weights were due to solid tumor growth and not merely to an influx of inflammatory cells (Fig. 3C). Thus, only the combination of Ad5mTRAIL+CpG was effective at controlling renal tumor outgrowth.

Both CD4 and CD8 T cells can mediate tumor regression [16], [37], and the relative contribution of each lymphocyte population may vary depending on the tumor model. To identify the contributions of CD4 and CD8 T cells in protective antitumor immunity that resulted from Ad5mTRAIL+CpG administration, we performed IR tumor challenges with parental Renca cells as above, then depleted mice of either CD4 or CD8 cells prior to giving immunotherapy on d 7. Depletion of CD8 cells prior to Ad5mTRAIL+CpG therapy resulted in a significant increase in primary renal tumor size (Ad5mTRAIL+CpG vs Ad5mTRAIL+CpG – CD8, *p* = .002), indicating that in the absence of CD8 cells, Ad5mTRAIL+CpG therapy was no longer effective (Fig. 3B). Depletion of CD4 cells led to a slight increase in primary renal tumor size as compared to non-depleted mice, but this difference was not statistically significant (*p* = 0.14). The latter finding is in contrast to our previous results in the s.c. Renca model, wherein CD4 depletion actually enhanced the efficacy of Ad5mTRAIL+CpG therapy [16].

Both gross and histological examination of kidneys showed that PBS-treated mice developed bulky renal tumors that obliterated normal kidney structure and greatly increased the overall kidney size (Fig. 3C). In contrast, kidneys from mice that received Ad5mTRAIL+CpG retained much of their normal appearance and architecture. Small areas of renal tumor growth were still present at this time, however, indicating that tumor eradication was ongoing (arrows, Fig. 3C). Kidneys from mice that were depleted of CD8 cells prior to Ad5mTRAIL+CpG therapy resembled those of the PBS group; bulky renal tumors were evident that consumed much of the normal kidney architecture and increased the overall organ size. Collectively, the data in Figure 3 demonstrate that local administration of Ad5mTRAIL+CpG immunotherapy initiated a CD8-dependent immune response that was able to eradicate previously established renal tumors in mice.

### IR administration of Ad5mTRAIL+CpG generates systemic immune responses

**Figure 4:**
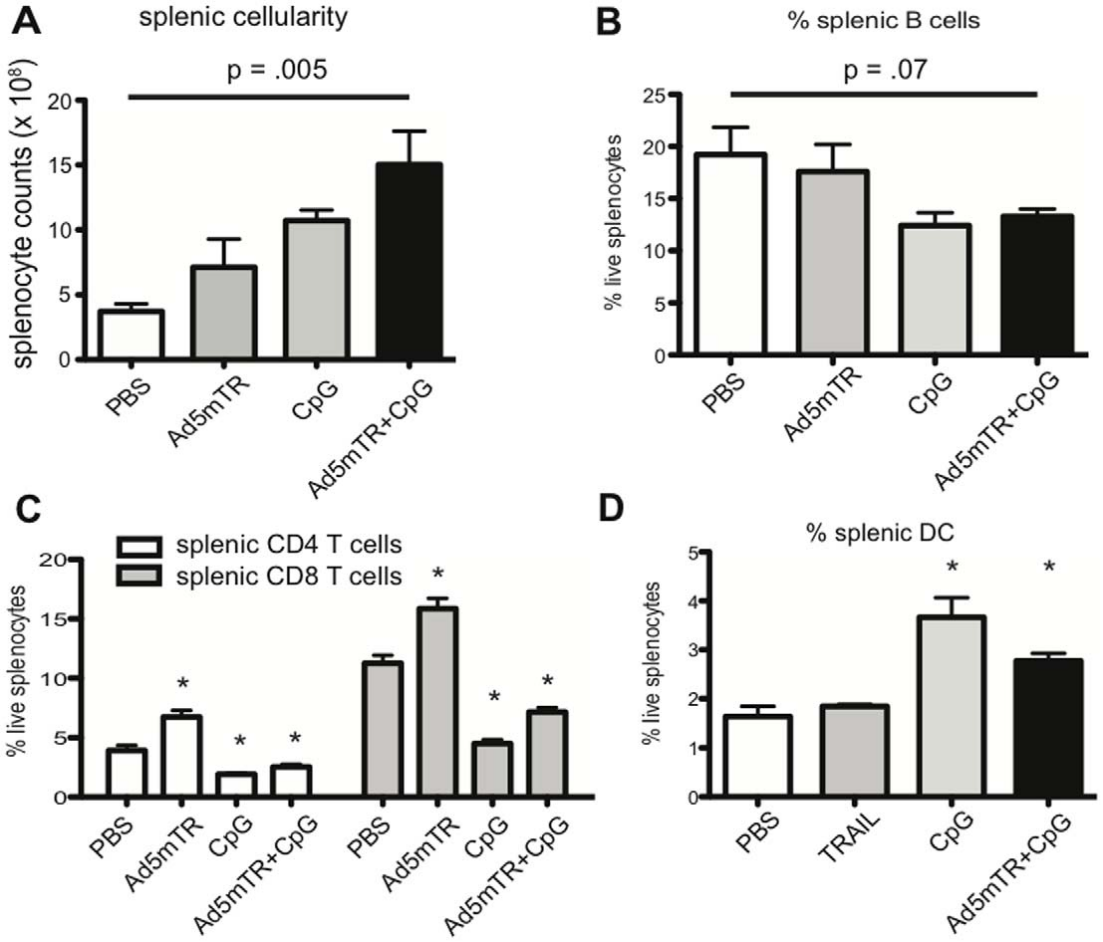
IR administration of Ad5mTRAIL+CpG generates systemic immune responses. (A) Parental Renca cells were injected IR, followed by administration of the indicated therapies IR on d 7. Spleens were excised and live cells counted on d 12. Mean live splenocyte counts from 1 experiment, representative of 4, are shown (n = 4 mice per group). For PBS vs Ad5mTRAIL *p* = 0.17; for PBS vs CpG *p* = <0.001; for Ad5mTRAIL+CpG vs Ad5mTRAIL *p* = 0.06; for Ad5mTRAIL+CpG vs CpG *p* = 0.16. (B–D) Flow cytometric analyses of spleens are shown; numbers indicate the percentages of live splenic B cells (B), CD4 and CD8 T cells (C) or CD11c^high^ DC (D) (* indicates *p*<0.05 versus PBS controls).

Immune-based therapies are promising treatment options for metastatic cancer as both effector and memory T cells have the potential to traffic systemically and provide protection against disseminated tumor outgrowth. As a next step in evaluating the potential utility of Ad5mTRAIL+CpG for metastatic RCC, we examined mice for evidence of a systemic immune response following local administration of therapy at the site of primary renal tumor growth. Mice were challenged IR with parental Renca cells, then treated IR with PBS, Ad5mTRAIL alone, CpG alone, or Ad5mTRAIL+CpG on d 7. Mice receiving either Ad5mTRAIL+CpG or CpG alone developed splenomegaly by d 12 (Fig. 4A; Ad5mTRAIL vs PBS, *p* = 0.17; CpG vs Ad5mTRAIL+CpG, *p* = 0.16), which remained through d 23–25 (data not shown). To determine the extent to which different splenocyte cell populations were specifically expanded in Ad5mTRAIL+CpG-treated mice, we evaluated the frequencies of splenic B220^+^ B cells, CD3^+^CD4^+^ T cells, CD3^+^CD8^+^ T cells, CD11c^high^ DC, CD11b^+^ macrophages, and DX5^+^ NK cells. Splenic B cell, CD4 T cell, and CD8 T cell frequencies actually decreased in Ad5mTRAIL+CpG-treated mice relative to PBS controls (Fig. 4B and C). However, due to the increase in splenic cellularity, equal or greater overall numbers of these cells were present in Ad5mTRAIL+CpG-treated mice (data not shown). The only cell population that did show a statistically significant increase in frequency, compared to PBS-treated mice, was the CD11c^high^ splenic DC compartment (Fig. 4D and data not shown).

**Figure 5:**
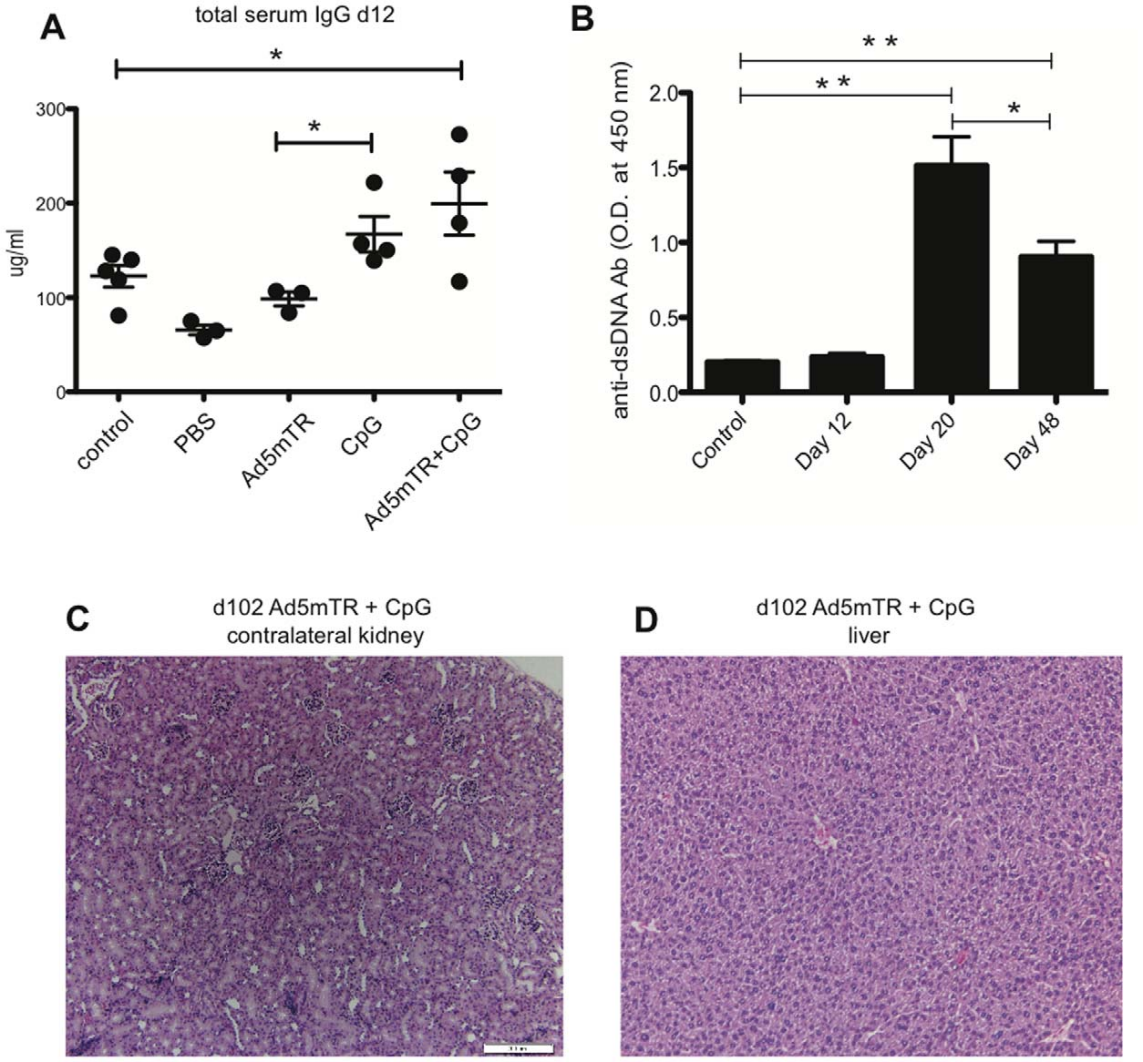
Ad5mTRAIL+CpG induces humoral immunity without autoimmunity. (A) Total serum IgG concentrations obtained on d 12 from the indicated treatment groups. Only Ad5mTR+CpC resulted in a significant increase in serum IgG versus tumor-free control mice. (B) Anti-dsDNA serum concentrations obtained on d 12, 20, and 48 from Ad5mTR+CpG-treated mice. (For both A and B, * indicates *p*, 0.05; ** indicates *p*<0.01). (C, D) Representative photomicrographs of H&E stained sections of the contralateral kidney (C) and liver (D) taken from one Ad5mTR+CpG-treated mouse (of a total of 7 analyzed) at d 102 after IR tumor challenge. Scale bar indicates 200 µm.

CpG1826 can activate B cells [13], [14], and although we did not detect a specific expansion in the B cell compartment, we examined the possibility that Ad5mTRAIL+CpG treatment induced a humoral immune response. Mice were bled on d 12 after tumor challenge, and serum IgG levels were tested by ELISA. At this timepoint, Ad5mTRAIL+CpG-treated mice showed statistical increases in serum IgG relative to control tumor-free mice (*p* = 0.048), whereas CpG-treated mice did not (*p* = 0.07; Fig. 5A). CpG treatment did increase serum IgG relative to mice that received Ad5mTRAIL alone (*p* = 0.03). An examination of total serum IgG at d 20 revealed an even more pronounced augmentation in Ad5mTRAIL+CpG-treated mice (data not shown). Adenovirus-mediated gene therapy leads to the formation of anti-adenovirus Ab [27], so we also examined the serum obtained on d 12 and d 20 after tumor challenge for the presence of anti-adenovirus IgG. Administration of Ad5mTRAIL+CpG led to a striking increase in serum anti-adenovirus IgG, which resulted in an approximately 8-fold elevation at d 12, and an approximately 11,000-fold elevation at d 20, relative to PBS-treated control mice (data not shown).

Previously, two reports had indicated that tumor challenge of mice leads to the formation of anti-dsDNA Ab, some of which can have tumoricidal effects in vitro and in vivo [38], [39]. To determine the extent of anti-dsDNA Ab production after Ad5mTRAIL+CpG administration for IR Renca tumor challenge, we measured the amount of anti-dsDNA Ab in the serum by ELISA. At d 12 the amount of anti-dsDNA Ab present in serum was low; however, the relative amounts increased by d 20 then declined by d 48 (Fig. 5B). Thus, administration of Ad5mTRAIL+CpG after IR Renca tumor challenge induces a humoral immune response marked by increases in serum IgG, which is composed of both anti-adenoviral and anti-dsDNA Ab.

Given the presence of increased amounts of circulating Ab in Ad5mTRAIL+CpG-treated mice, it was possible that immune complex formation/deposition could lead to subsequent autoimmunity. To explore this possibility, we challenged mice with parental Renca, and administered Ad5mTRAIL+CpG on d 7. As this was the only treatment group that had shown a significant increase in total serum IgG levels, the other treatment groups were not examined. At d 102, livers, contralateral kidneys, and tumor-challenged kidneys from surviving mice (n = 7) were examined for pathological evidence of autoimmunity. Importantly, there was no sign of immune-mediated pathology in the contralateral kidneys and livers (Fig. 5C and D). At this late timepoint, cavitary lesions surrounded by areas of interstitial lymphocytic inflammation were present in several of the tumor-challenged kidneys; these changes were consistent with tumor elimination (data not shown). Thus, although Ad5mTRAIL+CpG was administered at the site of primary renal tumor growth, this therapy induced systemic immune changes marked by splenomegaly and increased serum IgG that did not lead to autoimmunity.

### IR Ad5mTRAIL+CpG immunotherapy stimulates a CD8-dependent eradication of metastatic RCC

**Figure 6:**
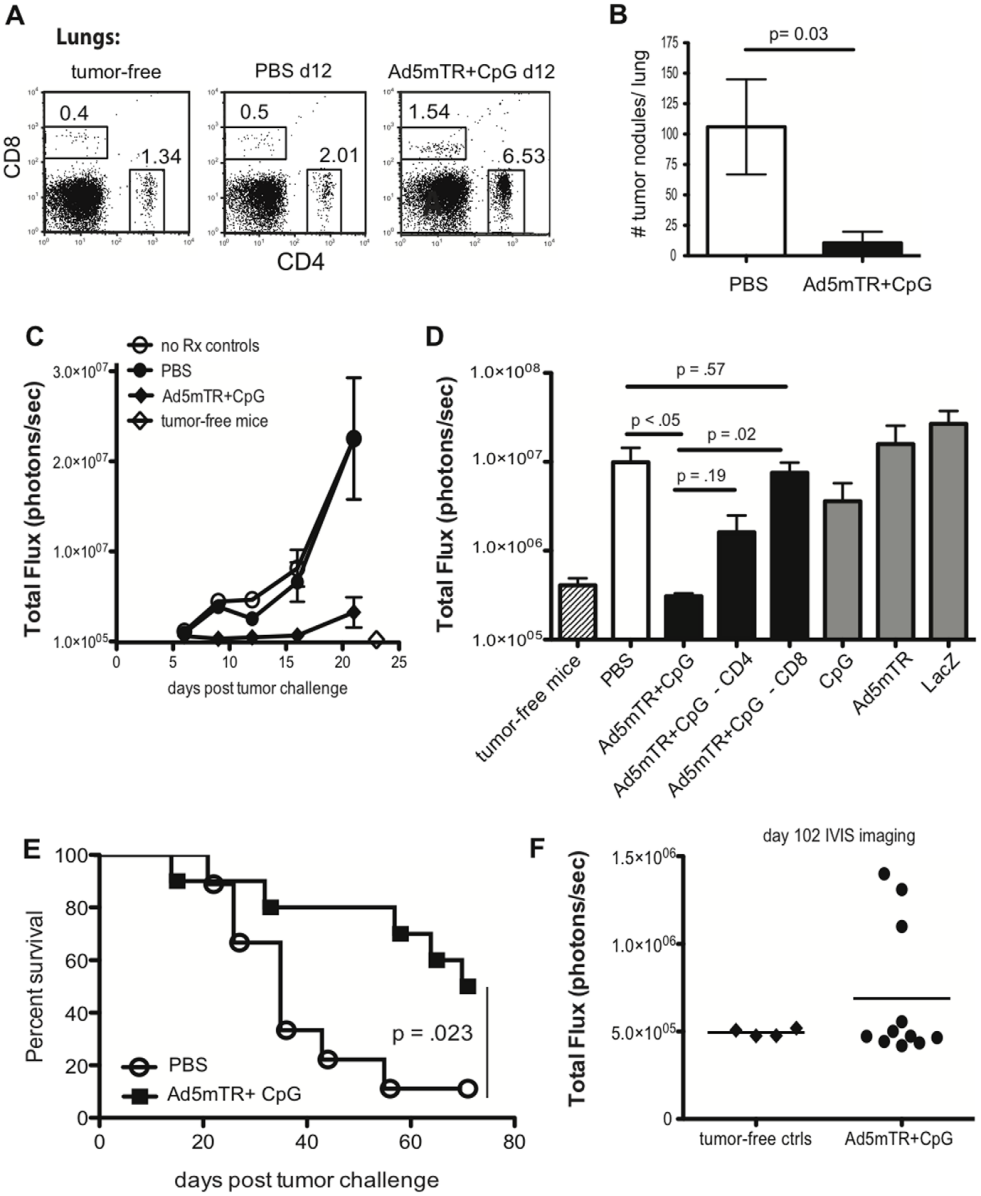
IR administration of Ad5mTRAIL+CpG stimulates CD8-dependent eradication of metastatic Renca tumors. (A) Parental Renca cells were given IR, followed by administration of PBS or Ad5mTR+CpG IR on d 7. Flow cytometric analyses examining T cell infiltration were performed on excised lungs on d 12. Numbers indicate the frequencies of live cells within the gated regions. (B) Mice were challenged as in (A), followed by PBS or Ad5mTR+CpG on d 7. Lungs were excised on d 21, and surface lung nodules were enumerated as in Figure 2 (n = 15 mice per group, combined from 3 independent experiments). (C, D) Renca-LUC cells were given IR, followed by administration of PBS or Ad5mTR+CpG IR on d 7. (C) Mean total light flux values from 5 mice per indicated treatment group are shown from d 6–21. The mean light flux value for 5 tumor-free mice is shown at d 23 to denote background level of detection. Light flux values for Ad5mTR+CpG-treated mice at d 21 are statistically insignificant to those from tumor-free mice (*p* = 0.182), suggesting tumor eradication was nearly complete. Ad5mTR+CpG vs PBS at d 21, *p* = 0.032. (D) Mean total light flux values measured on d 21 for 3–10 mice per group, combined from 2 individual experiments, are shown. Ad5mTR+CpG vs Ad5mTR+CpG with CD4 depletion *p* = 0.186; Ad5mTR+CpG vs Ad5mTR+CpG with CD8 depletion *p* = 0.015; Ad5mTRAIL+CpG – CD8 vs Ad5mTRAIL+CpG – CD4 *p* = .037; PBS vs Ad5mTR+CpG with CD8 depletion *p* = 0.573; PBS vs CpG *p* = 0.440. (E) Mice were challenged as in (A), followed by PBS or Ad5mTR+CpG on d 7. Survival data from 10 mice per group are shown through d 72. (F) Mice were challenged on d 0 with Renca-Luc, treated on d 7 with Ad5mTRAIL+CpG, then re-examined at d 102 by BLI for signs of tumor recurrence. Of 15 tumor-challenged mice, 11 survived to d 102, and 8 of these showed no evidence of tumor re-growth.

The primary clinical application of immunotherapy for RCC is in the clearance of metastases, rather than localized tumors. To evaluate the ability of local (IR) Ad5mTRAIL+CpG administration to clear metastatic tumor burdens, mice were given an orthotopic tumor challenge with parental Renca, followed by IR Ad5mTRAIL+CpG therapy or PBS on d 7. The lungs were examined by flow cytometry on d 12 to determine the extent to which Ad5mTRAIL+CpG therapy increased the frequency of CD4 or CD8 T cells at this site of metastasis. Mice that received Ad5mTRAIL+CpG showed increased frequencies of both CD4 and CD8 T cells in the lungs (Fig. 6A). In a second set of mice, manual enumeration of surface lung tumors at d 21 revealed a significant decrease in the number of tumor nodules present in mice that received Ad5mTRAIL+CpG compared to PBS-treated mice (Fig. 6B). We then performed similar experiments using IR injection of Renca-Luc cells. While it was not possible to measure individual kidney versus lung tumor burdens in live mice via BLI for technical reasons, it was possible to determine the total tumor burden per mouse. Using this technique, we found that Ad5mTRAIL+CpG treatment led to a marked reduction in body-wide tumor outgrowth, as compared to PBS treatment, that was evident within days of administering immunotherapy (Fig. 6C). Ad5mTRAIL+CpG treatment on d 7 resulted in a Luciferase signal at d 21 that was no higher than that observed in tumor-free mice (*p* = 0.078), indicating that the total body tumor burden had been reduced to the background level of detection at this time point (Fig. 6C). Pooled data from multiple experiments produced a similar result at d 23 (Ad5mTRAIL+CpG vs tumor-free control mice, *p* = 0.14) (Fig. 6D). Moreover, neither CpG alone, Ad5mTRAIL alone, nor a control adenovirus were able to bring about a significant reduction in body-wide tumor outgrowth (Fig. 6D). Thus, optimal tumor regression required both Ad5mTRAIL and CpG, and was not due to non-specific effects of IR adenovirus administration. As before, we then determined the extent to which CD4 and/or CD8 T cells were required for this protective effect by depleting mice of these cell populations prior to administration of Ad5mTRAIL+CpG. Similar to what was observed for primary renal tumor regression, we found that mice lacking CD8 T cells were unable to control metastatic RCC tumor outgrowth following Ad5mTRAIL+CpG therapy, resulting in large tumor burdens similar to those seen in PBS-treated mice (Fig. 6D). Depletion of CD4 T cells had an intermediate effect that was not significant.

Finally, we determined the extent to which IR Ad5mTRAIL+CpG administration resulted in a long-term survival advantage for mice with primary and metastatic renal tumors. Parental Renca cells were injected IR, and Ad5mTRAIL+CpG or PBS was given locally on d 7. Therapeutic intervention led to a significant survival benefit that was evident through d 72 (Fig. 6E). In a separate experiment, we challenged mice IR with Renca-Luc, treated on d 7 with Ad5mTRAIL+CpG, and examined mice for signs of tumor re-growth through d 102. Here, 11 of 15 mice survived to d 102, and of these only three showed signs of tumor recurrence by BLI, with 8 remaining tumor-free (Fig. 6F). Thus, local administration of Ad5mTRAIL+CpG at the primary renal tumor site induces protective anti-tumor immunity that leads to the systemic eradication of disseminated tumors in a murine model of metastatic RCC.

## Discussion

The clinical management of RCC is highly variable, and depends on tumor staging at the time of diagnosis. For localized renal tumors with no evidence of metastases or with a single metastasis, radical nephrectomy with surgical resection of the metastatic lesion is the standard therapeutic approach, and 5-year survival rates for these patients are high [40]. However, nearly 30% of patients have multiple metastases at diagnosis, and an equal percentage will develop metastatic tumor recurrence following nephrectomy. Metastatic RCC is largely thought of as an incurable disease, with a median survival time of only 18 months [1]. Administration of multikinase inhibitors such as sunitinib and sorafenib, or antibodies against vascular endothelial growth factor (VEGF) receptors are largely palliative options, since complete remissions in response to these agents are rare [41]. In a small subset of patients the combination of radical nephrectomy plus high-dose IL-2 can be curative, but this approach is contraindicated in most individuals due to the severe toxicities associated with IL-2 administration [3], [4], [5]. Shortcomings in current therapeutic options provide the rationale for continued attempts to identify novel treatment options for patients with metastatic RCC. At the same time, durable responses to IL-2 therapy illustrate that immunotherapy can be effective, and suggest that less-toxic immunotherapies, given either with or without radical nephrectomy, could be beneficial for a greater number of patients.

In our current study, we used a single IR administration of Ad5mTRAIL+CpG to induce protective T cell immunity against metastatic RCC. Of note, the adenoviral vector we used is replication-deficient and encodes a membrane-bound form of murine TRAIL. Consequently, we expected only limited direct killing of tumor cells within the kidney due to the lack of dissemination of the vector-derived TRAIL protein or the vector itself via replicative spread. Despite these limitations, IR Ad5mTRAIL+CpG injection gave rise to systemic T cell responses that were needed to fully suppress local and metastatic tumor outgrowth, as well as a humoral immune response characterized by elevated total serum IgG, anti-adenovirus IgG, and anti-dsDNA Ab. To our knowledge, this is the first time that local administration of adenoviral-encoded TRAIL has been shown to elicit systemic immune responses in an orthotopic, spontaneously metastasizing tumor model. Other reports investigating the efficacy of TRAIL-based therapies against localized or metastatic tumors have been published, but these have focused on examining the direct tumoricial activity of the therapy when administered locally or systemically (such as i.v. injection of agonistic TRAIL antibodies [20]). It is important to keep in mind that there are strengths and weaknesses to all experimental tumor models used depending on the specific question(s) being asked; we were particularly interested in evaluating the contributions of the systemic antitumor immune response induced subsequent to Ad5mTRAIL+CpG administration to the overall control of the tumor burden.

As both Ad5humanTRAIL and CpG have shown minimal toxicity as single agents in Phase I clinical trials, they are excellent candidates for antitumor immunotherapies [9], [15]. We explored the potential of Ad5mTRAIL+CpG as a stand-alone therapy when the primary renal tumors were still quite small, but tumors cells had already spread to the lungs (Fig. 2E). It was important to demonstrate that tumor metastasis to the lung had occurred at the time of therapy, to eliminate concern that any decrease in lung tumor burden was due to prevention of metastasis, rather than clearance of developing lung metastases. However, in a clinical context, it is tempting to speculate that AdTRAIL+CpG could also be used as an adjunct therapy prior to nephrectomy in cases of advanced RCC. Thus, future studies examining the combination of surgery plus immunotherapy for treatment of larger tumors are warranted. Use of Ad5mTRAIL+CpG as an adjunct therapy to nephrectomy would require careful examination of the timing between immunotherapy administration and surgery, as TRAIL-induced apoptosis of tumor cells, apoptotic body uptake by local DC, and activation of naive tumor antigen-specific T cells would need to occur prior to removal of the tumor-bearing kidney.

Although the Renca tumor line has been widely used to model RCC in mice, other murine kidney cancer cell lines do exist [42]. These less-studied lines may have variable primary growth and/or metastatic properties that could influence the efficacy of experimental immunotherapies. Despite this fact, the majority of studies have used Renca administered either s.c., which only gives rise to localized tumors, or i.v., which produces experimental metastases [16], [30], [31], [32], [34], [43]. Much has been learned from studies using the s.c. and i.v. tumor challenge routes, but these models have important limitations. Consequently, use of the orthotopic Renca model is becoming more common, as this places primary tumors in the correct anatomical location that will spontaneously give rise to lung metastases [20], [21], [22]. As the lung is one of the primary sites of metastasis in human RCC [23], the orthotopic Renca model also provides another degree of clinical relevance that is lacking in other models. In addition, our use of Luciferase-expressing Renca cells represents an advance in the pre-clinical modeling of metastatic RCC, as it allowed us to sensitively and accurately detect tumor cells in the kidneys and lungs of mice as early as d 7 post-tumor challenge (Fig. 2D and E) and to quantitate the kinetic differences in total tumor burdens that occur in the presence and absence of immunotherapy (Fig. 5C).

The role of TRAIL in Renca tumor clearance has been investigated in several previous studies [16], [30], [44], and our current work complements and builds on these findings. For example, one study using an agonistic mAb against DR5 induced only minimal apoptosis of Renca cells *in vitro* and *in vivo* after i.v. tumor challenge, but robust *in vivo* protection was observed when combined with the proteasome inhibitor bortezomib [30]. Another study showed that while TRAIL impaired the growth of Renca hepatic metastases *in vivo*, it was not involved in the protective response against i.v.-induced Renca lung metastases [44]. Our data shows that administration of Ad5mTRAIL+CpG at the primary tumor site not only reduces the primary tumor burden, but also protects against the outgrowth of metastatic lung tumors (Fig. 5D and E). The difference between our finding and the previous reports may stem from the fact that the tumor challenge and treatment routes were different, leading to qualitatively different responses.

We showed previously that Ad5mTRAIL can bring about apoptotic cell death in Renca cells *in vitro* [45], and demonstrated the ability of Ad5mTRAIL+CpG to clear localized s.c. Renca tumors [16]. In the latter study, we found that depletion of CD4 T cells had a protective effect in mice that received Ad5mTRAIL+CpG, leading to prolonged survival relative to intact mice that received the same therapy. This finding suggested that regulatory CD4 T cells were suppressing CD8 T cell-mediated tumor clearance. In contrast, we now find that in mice with orthotopic Renca tumors, depletion of CD4 T cells in Ad5mTRAIL+CpG-treated mice results in larger tumor burdens (Fig. 3 and 5), indicating that CD4 T cells are directly contributing to tumor clearance and/or providing the necessary help to generate an optimal CD8 T cell response. These disparate findings imply that orthotopic tumor challenge and IR therapy administration give rise to fundamentally different tumor microenvironments and immune responses than what occur with standard s.c. tumor challenges.

In addition to being given as a therapeutic agent, TRAIL can be used by endogenous T cells as an effector mechanism to bring about tumor cell death. It was shown previously by Seki et al. that cytotoxic T lymphocytes did not use TRAIL or perforin to kill Renca tumor cells, but instead relied primarily upon FasL *in vivo* [34], particularly when the cognate antigen levels were low (determined with HA-expressing Renca-HA cells [46]). A preliminary assessment of cytolytic mechanisms in our system supported these findings, in that Ad5mTRAIL+CpG-mediated renal tumor clearance proceeded normally in mice that were lacking either TRAIL or perforin (data not shown).

Our current study demonstrates the feasibility of using T cell stimulation as a means to protect against primary renal and metastatic tumors in mice. Despite the fact that T cell-mediated eradication of advanced RCC has been documented in cancer patients, this is still a rare event [17], [18], [19]. RCC is an immunogenic tumor, yet most patients with advanced disease fail to respond objectively to immunotherapy. One explanation might be the inability of activated T cells to overcome tumor-derived immune suppression. Several reports have shown that the accumulation of tumor-induced suppressor cells, including myeloid-derived suppressor cells and regulatory T cells, is an impediment to the induction of protective antitumor immunity in RCC patients [47], [48], [49], [50] and in murine Renca models [31], [51], [52], [53]. We applied Ad5mTRAIL+CpG therapy to mice in which the primary renal tumors were left intact. This immunotherapy led to a striking reduction in body-wide tumor burdens through d 21–23 (Fig. 5), but we did observe tumor regrowth in approximately 50% of mice (Fig. 6E and 6F). It is possible that combining Ad5mTRAIL+CpG with low-dose cyclophosphamide or sunitinib to remove suppressive cell populations would lead to complete tumor eradication in a larger percentage of mice.

Our data demonstrate that combinatorial immunotherapy consisting of adenovirus-encoded TRAIL+CpG1826 in an orthotopic RCC model in mice was effective in inducing a systemic T cell response that contributed to reducing local and metastatic tumors. While a humoral immune response was also evident in treated mice, our studies were not designed to examine the contribution of the humoral response to the eventual reduction in tumor burden. The abrogation of tumor clearance after depletion of CD8^+^ cells would suggest that a protective anti-tumor humoral response was not present, but formal investigation would need to be done to confirm this conclusion. Perhaps more importantly, the transient increases in serum IgG and anti-dsDNA did not lead to development of autoimmunity, further implying that this immunotherapeutic approach has the potential for clinical translation. Our conclusion is supported by the two prior reports on tumor-induced anti-dsDNA Ab, in which it was suggested that these antibodies had tumoricidal functions in vitro and in vivo, and did not trigger autoimmunity [38], [39]. In particular, Ad5TRAIL+CpG may be a suitable treatment alternative for patients with inoperable, advanced RCC, in that IR administration of Ad5TRAIL+CpG may stimulate systemic cellular anti-tumor immunity that can target any residual primary tumor not directly killed by TRAIL as well as distal metastases.
